# Esophageal Wound Vacuum Placement for Anastomotic Leak: Lessons Learned From First Time Use at a Tertiary Care Center

**DOI:** 10.1097/PG9.0000000000000114

**Published:** 2021-08-05

**Authors:** Megan Glait, Jonathan Wong, Amornluck Krasaelap, Amy Wagner, Dave Lal, John Schneider, Diana Lerner

**Affiliations:** From the * Medical College of Wisconsin, Wauwatosa, WI; † Division of Pediatric Gastroenterology, Hepatology and Nutrition, Children’s Hospital of Wisconsin, Milwaukee, WI; ‡ Children’s Mercy Hospital Kansas City, Kansas City, MO; § Division of Pediatric Surgery, Children’s Hospital of Wisconsin, Milwaukee, WI.

**Keywords:** esophageal atresia, tracheoesophageal fistula, stricture

## Abstract

Esophageal atresia and tracheoesophageal fistula (TEF) are rare birth anomalies typically requiring corrective surgery over the first few months of life. Esophageal surgery can lead to a life-threatening anastomotic leak. Esophageal wound vacuums have seen increased use in adults and one cohort of children as a therapeutic modality. This case study explores a tertiary care pediatric hospital’s introductory experience in utilizing this technique. A 19-month-old male underwent staged repair for esophageal atresia/tracheoesophageal fistula requiring an esophageal stricture resection with primary anastomosis. An anastomotic leak was successfully managed with wound vacuums. Our experiences highlighted the need for individualized treatment plans with this therapy based on feeding capabilities, side effects of the vacuum, placement method, and replacement strategies.

**What Is Known**

- Esophageal leak is a life-threatening complication in the setting of esophageal anastomotic surgery;
- A range of modalities exist from conservative changes to invasive surgical correction;
- Negative pressure wound vacuum placement has shown promise as a therapeutic option for esophageal leak.

**What Is New**

- We recount initial experiences at a tertiary care children’s hospital in utilizing esophageal wound vacuums;
- Our experience informs strategies for possible variations in placement, advice on replacement, and factors to consider on an individual basis.

## INTRODUCTION

Esophageal atresia (EA) and tracheoesophageal fistula (TEF) affects 1 in 4000 births in the United States.^1^ Initial management includes primary anastomosis and, if a fistula is present, surgical separation of the trachea and esophagus. Approximately one-quarter of these patients will experience an anastomotic leak (AL).^2,3^ Options for treating ALs include conservative measures such as NPO status, nasogastric tube placement, and empiric antibiotics. More aggressive options include endoscopic closure, esophageal stent placement, or surgical exploration.^4^ Modern negative pressure wound vacuum-assisted closures (VACs) have been utilized since the early 1990s and have since shown to be an effective therapy option for superficial wound healing.^5–7^ More recently, the use of wound vacuums in adult upper gastrointestinal injuries has shown promise.^8,9^ A recent study by Manfredi et al demonstrated efficacy in a pediatric cohort of 17 patients with esophageal perforations. Wound VAC use for ALs was associated with a significantly better success rate (88%) as compared to stents (*P* = 0.032).^10^ Esophageal stents also have longer dwell times and known complications of migration, patient discomfort, and local pressure necrosis.^10^ We present our experience with initial use at a tertiary referral center involving wound VAC therapy for persistent esophageal ALs. Informed consent was obtained from the parents of both patients for publication of the case details.

## CASE REPORTS

**FIGURE 1.**
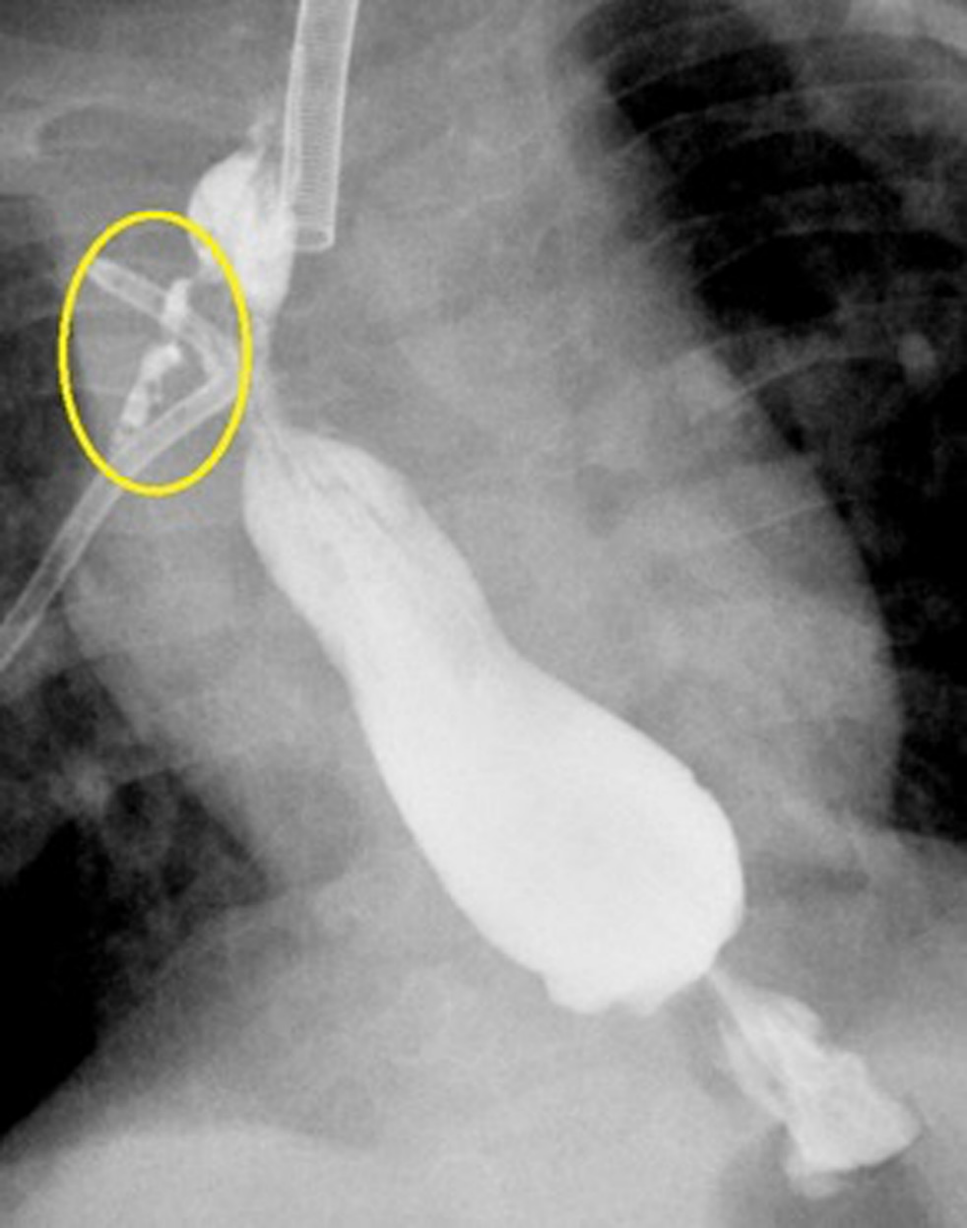
Esophagogram showing anastomotic leak prior to wound vacuum intervention.

**FIGURE 2.**
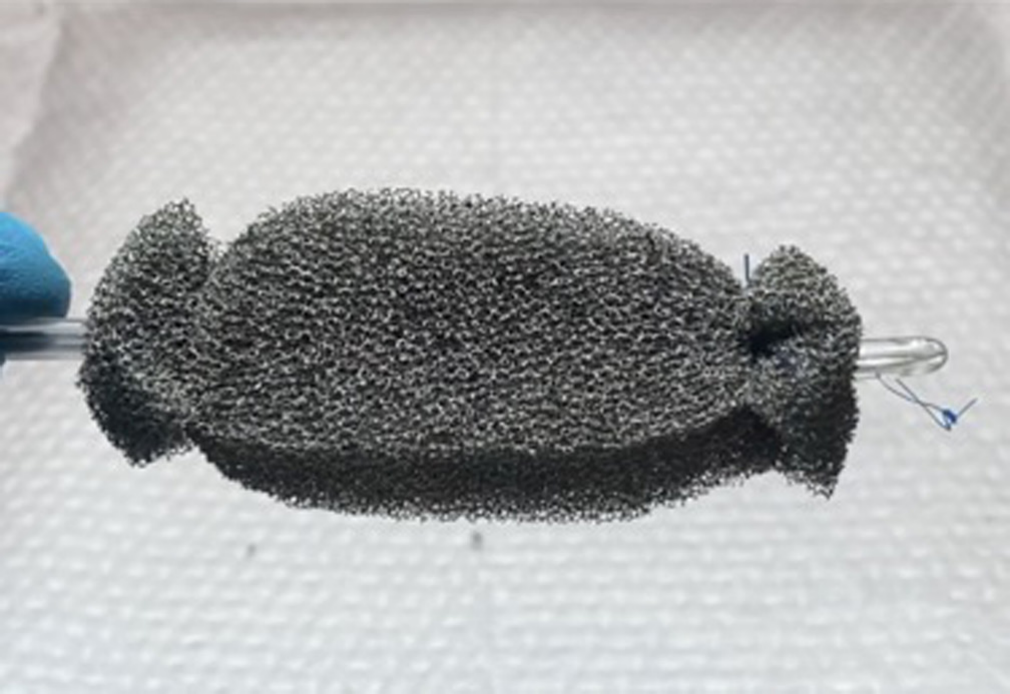
Photograph of completed wound vacuum device prior to placement.

**FIGURE 3.**
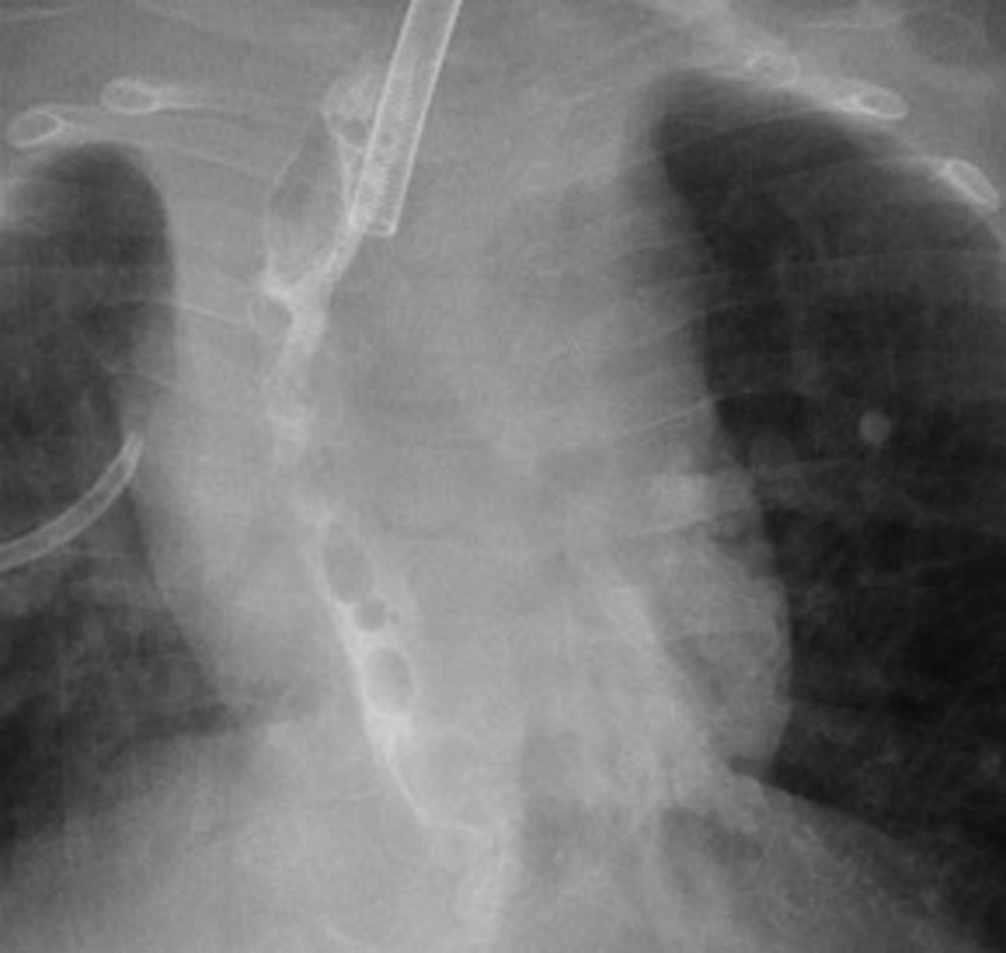
Esophagogram showing resolution of the anastomotic leak after wound vacuum intervention.

Our first patient was a 19-month-old male with a complex past medical history, including ex-27-week of gestation, multiple cardiac defects, bronchopulmonary dysplasia, and TEF/EA. Due to long-gap EA, the patient underwent a staged repair over several months involving esophageal retention sutures and magnetic compression anastomosis. Following this repair, the patient developed a persistent anastomotic stricture refractory to numerous balloon dilations, triamcinolone injections, mitomycin C applications, incisional therapies, and stent placements. Despite additional attempts to facilitate esophageal healing including a laparoscopic cruroplasty and Nissen fundoplication, the stricture persisted, requiring revision of his esophageal anastomosis. On postoperative day 7, an esophagogram was notable for an AL (Fig. 1). Conservative measures over the next two weeks failed to resolve the leak. A decision was made to attempt treatment of the leak with an esophageal wound VAC. The patient was taken to the operating room where a twelve French Salem Sump tube with an attached wound vacuum (Fig. 2) was pulled through the gastrostomy site under endoscopic visualization.^10^ Following placement, nutrition was maintained via a previously placed jejunostomy tube. A repeat endoscopy after three days of therapy showed an improved but persistent leak; therefore, the wound vacuum was replaced.^10^ During the second period with the wound VAC, the patient had episodes of emesis, agitation, and thick secretions requiring frequent suctioning. Repeat endoscopic evaluation with intraoperative esophagogram after a 4-day interval showed resolution of the leak and the device was removed (Fig. 3). The patient did well after removal and was discharged 3 days postprocedure (10 days after initial wound VAC placement).

## DISCUSSION

Using lessons learned from our first placement, a second case was performed in a 20-month-old female (also with TEF/EA) several months later with successful resolution of an AL.^11^ Our experience demonstrates the efficacy of the esophageal wound VAC technique for ALs with little previous experience. The two patients were similar in several aspects with both requiring wound VAC at approximately 1.5 years of age, ALs occurring after resection and re-anastomosis, and placement occurring via a retrograde approach. Furthermore, in both cases nutrition was given through preexisting jejunostomy sites, secretion management was an issue and 2 wound VACs were required for resolution and subsequent discharge.

The cases varied in that the first patient had his wound VACs in place for 3 and 4 days versus 5 and 6 days in the second. With regards to placement, the second patient had her sump tubing break upon retrograde pulling for the replacement of the first wound VAC.

These experiences emphasized that elements of the wound VAC technique are patient-specific; such as use of a retrograde versus anterograde approach, number of replacements, nutritional support during VAC placement, and possible complications. Equipment utilized included a 5.5 mm endoscope, 12Fr Salem Sump tubing, wound vacuum pump and sponge kit cut to size, 2-0 silk suture, and a needle driver. Both cases were performed in operating rooms in collaboration with surgical colleagues.

## LESSONS LEARNED

### Timing

Resolution of both ALs required 2 wound VAC placements and both ALs resolved after 7 and 11 days of placement, respectively. Although no standard has been established, current recommendations are to leave the device in place no longer than 4–7 days before endoscopic evaluation.^10^ Previous studies in adult patients have made similar recommendations noting wound VAC changes every 2–4 days were standard.^8^ In the previously referenced pediatric study of 17 patients, the average wound VAC dwell time was 5.5 days with a max of 8.^10^ Although it would be preferable to perform 1 procedure versus 2, this must be balanced with the risk of the sponge embedding into esophageal tissue. Future studies on total time to mucosal healing may justify longer wound VAC intervals in this regard.

### Assembly

**FIGURE 4.**
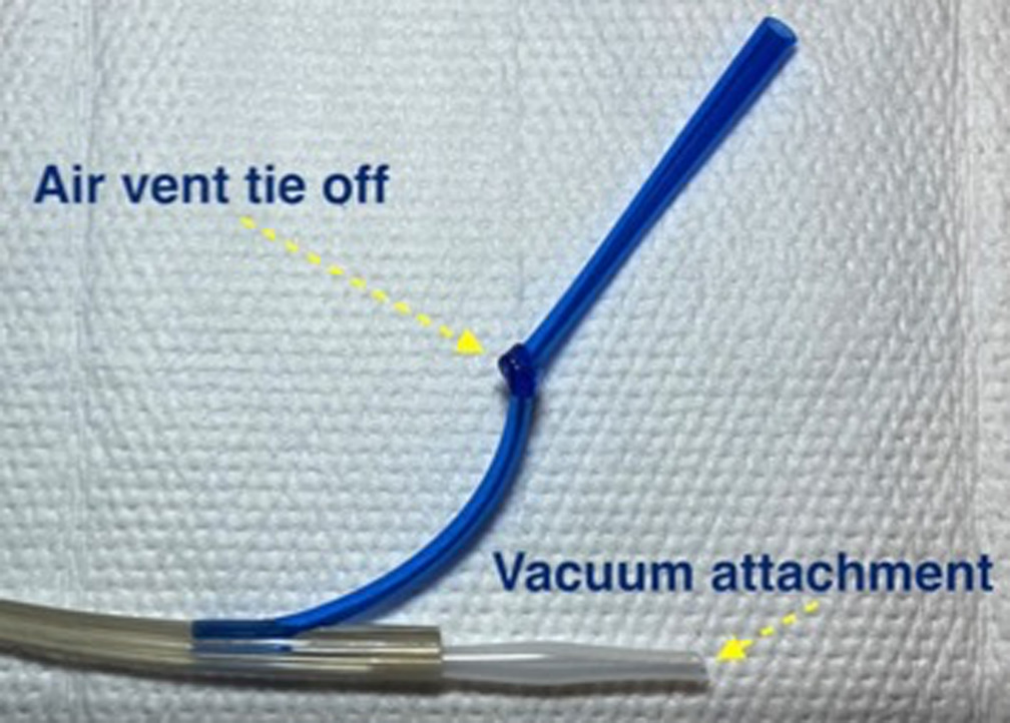
Photograph highlighting important aspects of esophageal wound vacuum device assembly.

Important technical aspects of this technique included proper sponge securement and physical placement on the Salem Sump tubing. Specifically, the sponge may slip if the ties on either end are not driven through the sump tubing itself. Adequate suction may not be possible unless all the outlets on the Salem Sump are circumferentially covered by sponge material. The air vent will require occlusion (tie off) and an adapter may be necessary to connect to the wound vacuum tubing (Fig. 4). As noted by Manfredi et al,^10^ when utilizing a retrograde approach, assembly of the wound VAC device should occur after the tube has been withdrawn through the mouth as in most instances, the device will be larger than the gastrostomy site. We found it helpful in some cases to pass a wire and insert the wound VAC over the wire. Fluoroscopic guidance can be helpful to ensure the entire VAC assembly is positioned correctly and spans the length of the esophageal leak. It is necessary to change the Salem Sump tubing with each wound VAC change to prevent tube breakage from degradation of the tubing.

### Secretion management

Clinical symptoms appeared to be VAC placement dependent, highlighting the importance of proper esophageal location as well as the customization of the sponge itself. As noted by Manfredi et al, ^10^ initial settings of the wound vacuum should be continuous (125 mm Hg), but can be switched to intermittent (5 minutes on and 2 minutes off) if the patient demonstrates intolerance of secretions. Previous studies did note alternative pressure settings between 75 and 200 mm Hg but as these were not pediatric-specific, we opted to utilize 125 mm of Hg at continuous moderate intensity.^8,12^ This variation in technique, along with bedside oropharyngeal suctioning, proved efficacious in both cases.

## CONCLUSION

Esophageal wound vacuum placement is a useful modality for treating persistent esophageal leaks after failed conservative management. The described case was performed by providers with extensive experience in providing care for TEF/EA patients but with no prior esophageal wound VAC experience and no specialized equipment was utilized. While further studies are needed to evaluate superiority over traditional methods such as stents, we hope this publication along with a recently published video abstract (https://www.thieme.de/de/q.htm?p=opn/cs/20/11/13082874-289b158f) will assist in proper execution of this developing technique.^11^
